# Hyaluronan-enriched transfer medium (HETM) can improve the implantation rate in morphologically poor euploid blastocyst transfer

**DOI:** 10.1007/s00404-023-07083-9

**Published:** 2023-05-31

**Authors:** Koji Nakagawa, Takashi Horikawa, Yuji Orita, Emi Yamashiro, Hideaki Watanabe, Asako Shirai, Souichi Ogata, Hisayo Kataoka, Keiji Kuroda, Satoru Takamizawa, Rikikazu Sugiyama

**Affiliations:** 1 Center for Reproductive Medicine and Implantation Research, Sugiyama Clinic Shinjuku, 1-19-6, Nishi-Shinjuku, Shinjuku-Ku, Tokyo, 160-0023 Japan; 2 Takeuchi Ladies Clinic, Aira City, Kagoshima, Japan; 3 Vitrolife Japan, K.K., Minato-Ku, Tokyo, Japan

**Keywords:** Euploid blastocyst, Hyaluronan, HETM, Morphologically poor blastocyst, Pregnancy rate

## Abstract

**Purpose:**

Hyaluronan-enriched transfer medium (HETM) could improve the clinical pregnancy rate (CPR) for patients with repeated implantation failures (RIF). In contrast, there have been seldom reports addressing the potentially beneficial effects of HETM for morphologically poor blastocysts (MPBLs). Our study aimed to evaluate whether the use of HETM would improve the CPR for the patients who were transferred with euploid MPBLs.

**Methods:**

Patients who underwent single euploid blastocyst transfer between July 2020 and June 2022 were enrolled. We included only those blastocysts confirmed as euploid by PGT-A, and those blastocysts were transferred after thawing. The natural ovulatory cycle or hormone replacement cycle (HRC) protocol were used for endometrial preparation for frozen embryo transfer (FET). A total of 1,168 FET cycles were performed in the study period, including 954 cycles of morphologically good blastocysts (≥ 4BB in Gardner’s classification), and 85 cycles of MPBLs, of which 47 were transferred using HETM in FET (the HETM group), and the remaining 38 were transferred with the medium without hyaluronan (the control group). We compared the CPR between these two groups.

**Results:**

The characteristics of patients were similar between the HETM and control groups. The CPR in the HETM group was significantly higher than the control group (47.4% and 21.5%, respectively, p = 0.019). The multiple logistic regression analysis found that the use of HETM was a predictive factor of positive pregnancy outcomes (OR = 5.08, 95% CI = 1.62–16.0, p = 0.019).

**Conclusion:**

Our data suggests that HETM used in the euploid blastocyst transfer can improve the clinical pregnancy rates of morphologically poor blastocysts.

## What does this study add to the clinical work

**Table:** 

Hyarolunan-enriched transfer medium can make the morphologically poor euploid blastocyst help to implant.	

## Introduction

Modern assisted reproductive technology (ART) treatments consist of various processes, starting from ovarian stimulation, oocyte pick-up, insemination, embryo culture and finally embryo transfer. Hyaluronan-enriched transfer medium (HETM) has been clinically used in many ART clinics as a medium specially formulated for embryo transfer. The first report in 2002 on improved implantation rates of human embryos in ART treatment [1] was followed by many other RCT studies demonstrating the efficacy of HETM in human embryo transfer [2–4]. Urman et al. reported that HETM was effective in improving the implantation rates in a fresh embryo transfer cycle for patients with a history of one or more implantation failures and patients of advanced maternal age [4]. Our group has also previously reported that the clinical pregnancy rates of patients with repeated implantation failures (RIF) were improved in frozen embryo transfer cycles (FET) using HETM [5]. Based on this experience, our group has used HETM as the transfer medium for those patients with applicable histories or indications, or those who made a specific request after having an adequate consultation with the clinicians. A Cochrane review was recently published in 2020, which cited data from a number of clinical studies using HETM in embryo transfer. The Cochrane review supported the addition of hyaluronic acid (HA) in embryo transfer medium to improve the clinical outcomes of ART treatments, including improvements in live births per ART treatments and decreasing rates of miscarriage [6]. Interestingly, we noted that many previous studies had selected embryos with good morphological grades for transfer, because in general poor morphological grades are considered to give lower implantation potential.

More recently, preimplantation genetic testing for aneuploidy (PGT-A) is becoming commonplace, and it has been widely implemented in the treatment for patients with RIF or recurrent pregnancy losses, to reduce the chance of another failure in embryo transfer. While blastomere biopsy used to be a common procedure for PGT-A, nowadays it has been replaced almost entirely with trophectoderm (TE) biopsy at blastocyst stage [7]. In Japan, the selection of euploid blastocysts for transfer following PGT-A has been allowed since 2019. Recently, we reported that morphologically good blastocysts had higher clinical pregnancy rates than morphologically poor blastocysts even when we only selected euploid blastocysts for transfer [8]. However, it is still an open question if HETM could improve implantation and clinical pregnancy rates of blastocysts with poor morphological grades.

Therefore, we designed our study to investigate the effect of using HETM on clinical pregnancy rates (CPR) in patients who underwent euploid blastocyst transfer with poor morphological grade in a FET cycle. By transferring only euploid blastocysts by PGT-A, our study specifically addresses the relationship between embryo morphology and the transfer success rates in the presence of high concentration HA in embryo transfer medium.

## Materials and methods

### Study design

**Fig. 1:**
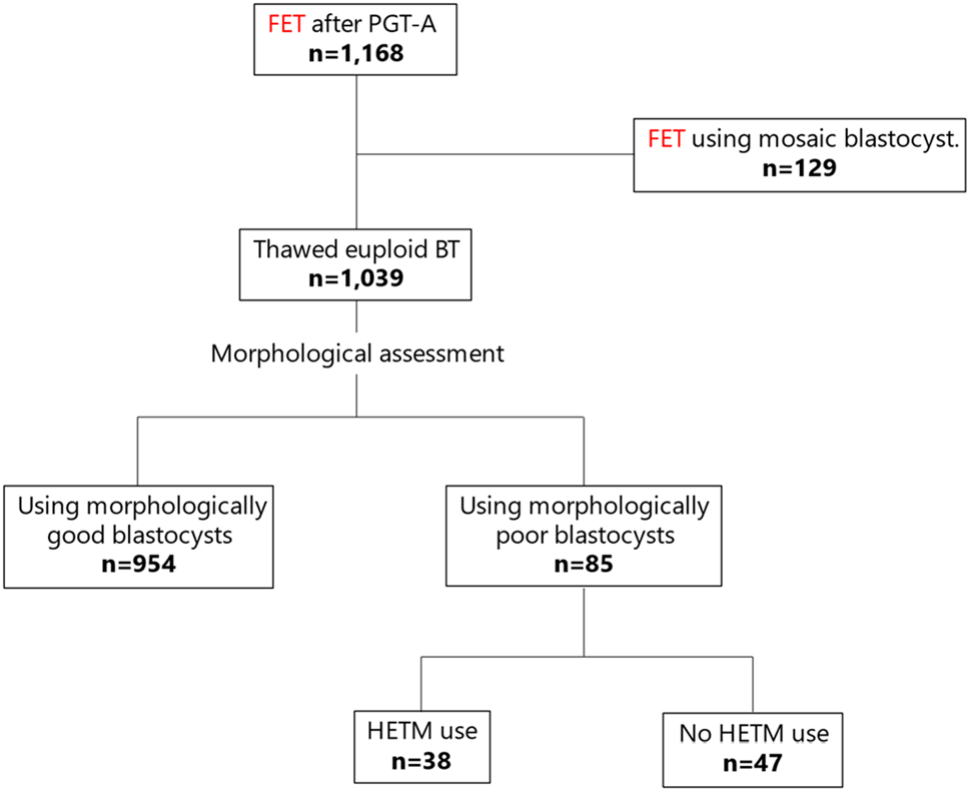
The selection flowchart of FET cycles in this study. In total 1168 FET were performed after PGT-A, which included 1039 FETs with euploid blastocysts. All transferred embryos had been subjected to morphological assessments after warming and prior FET, which resulted in 85 euploid blastocysts with poor morphological grades for FET. Among these, 38 were prepared with HETM just before the transfer, while the remaining 47 were prepared with the control medium. *FET* frozen embryo transfer, *PGT-A* preimplantation genetic testing for aneuploidy, *HETM* hyaluronan-enriched transfer medium

We conducted a single center, cross-sectional study between July 2020 and June 2022, which recruited a total of 1,168 FET with a single blastocyst at Sugiyama Clinic Shinjuku, Tokyo, Japan. A flowchart of the patient selection process is shown in Fig. 1. All blastocysts used in this study were diagnosed as euploid or mosaic based on PGT-A results. Those blastocysts showing 20% or less of mosaicism or segmental mosaicism were diagnosed as euploid and accepted for FET [9]. One hundred twenty-nine FET were performed with mosaic blastocysts, while the remaining 1039 FET were performed with euploid blastocysts. All transferred blastocysts were subjected to morphological assessments after thawing, which resulted in 954 blastocysts with good morphological grades and 85 blastocysts with poor morphological grades available for FET. Of the 85 morphologically poor euploid blastocysts, 47 were cultured and transferred in HETM and the remaining 38 were transferred in standard culture medium. Morphologically good blastocysts were defined as Gardner’s classification 4BB or better [10], while morphologically poor blastocysts were those with a C grade in either trophectoderm or inner cell mass (ICM). Throughout the entire study period (between July, 2020 and June, 2022), the same laboratory protocols, incubators, and other instruments for embryo culture and handling were used.

A written informed consent was obtained from all participating patients regarding PGT-A and euploid or low-frequency mosaic (LFM) blastocyst transfer, in accordance with the corresponding approval by the Institutional Reviewer Board (19–006). All treatment cycles used autologous oocytes, and no donor oocyte were included. All participating patients had single euploid blastocyst transfer within one year from the date of the PGT-A report. Additional written informed consent was obtained from all participants to perform FET with HETM, in accordance with the corresponding approval by the Institutional Reviewer Board of Sugiyama Clinic for retrospective investigation of unidentified data (21–006).

All participants received vaginal ultrasound imaging, hysterosalpingography, and hysteroscopy, to exclude uterine abnormality, uterine myoma, endometrial polyps, and intrauterine adhesion before participating in the study. No participant showed glucose intolerance and thyroid dysfunction. Exclusion criteria included autoimmune disease, anti-phospholipid syndrome, and chronic endometritis.

### Ovarian stimulation, IVF and embryo culture

All patients received ovarian stimulation for ART treatment, following a previously described protocol [11]. Daily administration of 50–100 mg of clomiphene citrate (Clomid®, Fuji Pharm, Tokyo) or 2.5–5.0 mg of letrozole (Letrozol®, Sawai, Osaka) was started from the 2nd or 3rd day of the menstrual cycle or withdrawal bleeding for 7 days, and 200–300 mg of recombinant follicle-stimulating hormone (rec-FSH; Gonal-F®; Merck BioPharma, Tokyo) or highly purified human menopausal gonadotropin (hMG, HMG-Ferring®, Ferring Pharmaceuticals. Tokyo) was administrated every two days from the 3rd day of the menstrual cycle. On the 10th day of the same cycle, transvaginal ultrasound imaging and hormone analysis was performed to evaluate follicle growth. When the dominant follicles reached ≥ 20 mm in diameter, the patient was given a recombinant human chorionic gonadotropin (rec-hCG; Ovidrel®; Merck BioPharma, Tokyo) or gonadotropin-releasing hormone (GnRH) agonist or both as the maturation trigger [12]. Approximately half of the patients were stimulated with this protocol, while the other half were treated with a modified progestins-primed ovarian stimulation protocol (modified PPOS), which differed only in the administration of progestins (10 mg of medroxyprogesterone Acetate; Hysron®, Kyowo-Kirin, Tokyo) from 8th to the day of maturation trigger. Thirty-five to thirty-six hours after the maturation trigger, the patient underwent transvaginal oocyte retrieval with or without general or local anesthesia.

The retrieved oocytes were inseminated by either conventional insemination (IVF) or intracytoplasmic sperm injection (ICSI), depending on the diagnosis after sperm preparation or the patients’ characteristics. Piezo-ICSI method was used for all ICSI cases [13]. After confirmation of normal fertilization, fertilized oocytes were continuously cultured to the blastocyst stage in a time-lapse incubator (Geri™, Genea Biomedx, VIC, Australia) supplied with mixed gas (5% O_2_, 6% CO_2_, and 89% N_2_) [14]. All embryos were cultured individually in GEMS GERI MEDIUM™ (Genea Biomedx, VIC, Australia), from immediately after insemination until trophectoderm (TE) biopsy.

### TE biopsy, NGS, and blastocyst cryopreservation

TE biopsy samples were isolated in our laboratory by mechanical dissection technique without laser assisted hatching (LAH). Under continuous time-lapse monitoring, the expanded blastocysts with adequate number of TE cells were biopsied for PGT-A. First, a small hole was made in the zona pellucida of the embryo using an infrared diode laser (Saturn 5™ Active, Cooper Surgical, CT, USA), to induce blastocoele collapse. Next, a biopsy pipette (Kitazato, Shizuoka, Japan) was inserted through that small hole into the perivitelline space to aspirate TE cells. After TE aspiration, approximately 5–10 TE cells were excised by mechanical blunt dissection technique [15]. These procedures were performed with micromanipulation instruments while keeping the embryo in droplets of PGD biopsy medium (Global, LifeGlobal, USA). The isolated TE cells were washed twice in sterile phosphate-buffered saline (PBS) supplemented with 1% polyvinylpyrrolidone (PVP), then transferred into a 0.2 ml PCR tube containing 2.5 μL of PBS and kept immediately in  – 20 °C until the DNA analysis. All biopsy samples were sent to the laboratory of KITAZATO BIOLABORATORY, Tokyo for whole genome amplification (WGA) by Sureplex DNA Amplification System (Illumina, CA, USA). The amplified genome samples were analyzed using next generation sequencing (NGS) in Miseq System (Illumina, CA, USA), and then the Copy Number Variation (CNV) chart was generated by BlueFuse Multi Software (Illumina, CA, USA) [7]. Following biopsy, all remaining blastocysts were cryopreserved using Kitazato Vitrification Medium VT505 and Cryotop® (Kitazato Tokyo). [16, 17], in readiness for the PGT-A analysis reports and the subsequent ploidy status assessments (euploid, mosaic, and aneuploid).

### Thawed euploid FET with or without HETM

Prior to frozen embryo transfer (FET) with an euploid blastocyst, the patient underwent uterine endometrial preparation for either natural ovulatory cycle (NOC) or hormone replacement cycle (HRC) depending on the pattern of patients’ menstrual cycle. For patients undergoing the NOC protocol, FET was performed five days after ovulation, which was verified by both vaginal ultrasonography and the elevation of serum progesterone level (≥ 2 ng/ml). Dydrogesterone tablets (Duphaston®, Mylan EPD, Tokyo) were administrated at 30 mg per a day as luteal support, starting from the day after ovulation until the day of pregnancy test, and 125 mg of hydroxyprogesterone caproate (Progeston depot®; Fuji pharma, Tokyo) was injected once on the day of FET. For patients undergoing the HRC protocol, the uterine endometrium was prepared using both 1.25 mg of conjugated estrogen tablets (Premarin 0.625 mg®, Wyeth, Tokyo, Japan) and 2.88 mg transdermal estradiol patch (Estrana TAPE 0.72 mg®, Hisamitsu Pharmaceutical, Tokyo, Japan), which were started on the third day of the menstrual cycle or three days after withdrawal bleeding until the day of pregnancy test. 90 mg vaginal progesterone gel (OneCrinone®; Merck BioPharma, Tokyo) and 30 mg/day of dydrogesterone tablets were administered, starting from day 13 of menstrual cycle. FET was performed five days after commencement of progesterone treatment [5]. Transdermal estradiol patch, dydrogesterone tablets, and vaginal progesterone gel were continued until the day of pregnancy test, and after a positive pregnancy test was verified, the patient maintained the same treatment protocol until 9–10 weeks of gestation.

We prepared the embryo transfer dish the day before FET, in which 1.0 ml of the embryo transfer medium was taken to the transfer dish and equilibrated for more than 18 h in an incubator at 37 °C, 6% CO2, 5% O2 and 89% N2. EmbryoGlue® (Vitrolife, Sweden) was used for the HETM group while continuous single culture medium (CSC-C, Fuji Film, Japan) was used for the control group without additional hyaluronan.

On the day of FET, the cryopreserved euploid blastocysts were warmed and checked for viability. These blastocysts verified alive were initially cultured with culture medium (CSC-C Fuji Film, Japan) for 4–8 h in an incubator set at 37 °C supplied with a mixed gas of 6% CO2, 5% O2 and 89% N2. In some blastocysts LAH was performed after warming (Saturn 5™ ACTIVE; Cooper Surgical) before further culture. Blastocysts were assessed again for the morphological grades using Gardner’s Classification [10] just prior embryo transfer was performed. Blastocysts which remained a 4BB or better after warming were marked as morphologically good blastocysts, while those blastocysts with C grade in either trophectoderm or inner cell mass (ICM) were marked as morphologically poor blastocysts.

After the post-warm culture and morphology grading, the euploid blastocysts selected for transfer were placed in the transfer dish, prepared with equilibrated HETM or control medium, and incubated for another 30–60 min. A single selected blastocyst was replaced into the patient’s uterus transcervically using a soft catheter (Kitazato ET catheter, Kitazato Supply, Shizuoka, Japan) while being monitored under transvaginal ultrasonography.

### Clinical outcomes

The pregnancy test was performed 9 days after FET by measuring serum human chorionic gonadotropin (hCG), where a positive pregnancy test was defined by > 10 IU/ml of serum hCG. A clinical pregnancy was recognized when the development of a gestational sac (GS) was verified by transvaginal ultrasound imaging 16–21 days after FET. A miscarriage was defined when the fetal heartbeat was unable to be confirmed by transvaginal ultrasound imaging 9 weeks after gestation. The clinical pregnancy rate (CPR) was calculated from the number of FET cycles with confirmed GS and the total cycle number of FET. The miscarriage rate (MR) was calculated from the number of miscarriage cycles and the total number of clinical pregnancies.

### Statistical analysis

The data acquired in the clinic was statistically analyzed by Fisher’s exact test or non-parametric test. A multivariable logistic regression model was constructed in order to find independent risk factors and complications associated with the pregnancy outcomes after FET using morphologically poor euploid blastocyst, while controlling for confounders. Odds ratios (OR) and their 95% confidence interval (CI) were computed. A p-value of < 0.05 was considered statistically significant. All statistical analyses were performed with EZR (Saitama Medical Center, Jichi Medical University, Saitama, Japan), which is a graphical user interface for R (The R Foundation for Statistical Computing, Vienna, Austria). More precisely, it is a modified version of R commander designed to add statistical functions frequently used in biostatistics [18].

## Results

**Fig. 2:**
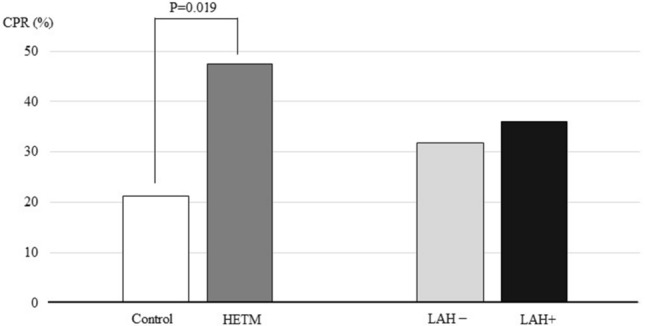
This graph indicates clinical pregnancy rates of the morphologically poor euploid blastocysts prepared with different conditions: HETM or control media, and the usage of LAH after warming of the embryos. The CPR of the HETM group was 47.4% [18/38] and significantly higher than that of the control group (21.3% [10/47], P = .019). In contrast, LAH did not influence the CPRs in the same settings (LAH + ; 36.0% [9/25] and LAH – ; 31.6% [19/60], p = 0.89). *HETM* hyaluronan-enriched transfer medium, *LAH* laser assisted hatching

Table 1 shows the clinical pregnancy, miscarriage and live-birth rates from transferring euploid blastocysts with either good or poor grades in the morphological assessment made after thawing. The patient characteristics in the study are shown in Table 2. The median age of the control and HETM groups were both 39.0 with no significant difference (p = 0.72). The proportions of advanced maternal ages patients (≥ 40 years old) were 40.4% and 42.1% in control and HETM groups, respectively, without significant difference (p = 1). The proportion of RIF patients in the HETM group was 50.0%, which was significantly higher than the control group (25.5%, p = 0.025), while the proportions of RPL patients were comparable (p = 0.447). The median number of previous ET attempts in the control and HETM groups were 1 and 2, respectively, but the difference was not statistically significant (p = 0.22). Conventional insemination method was used less frequently than ICSI (31.8% and 68.2%, respectively, p = 0.065), but we considered the difference was not significant enough to influence the overall outcomes. Regarding endometrial preparation before FET, the NOC protocol was selected for approximately half of the participants (56.5%), while the HRC protocol was selected for another half (43.5%) of the participants (p = 0.66). The proportions of patients that received LAH after thawing was also similar in both groups (29.8% in the control and 28.9% in the HETM group, respectively, p = 1.0). The clinical pregnancy rate (CPR) after FET of a morphologically poor euploid blastocyst was 32.9% (28/85) in this study, which was significantly lower than the CPR previously reported by Sato et al. (70.8% [17/24]) from transfer of a morphologically good blastocysts [19]. Among the FET with morphologically poor blastocysts, we found significant improvement in the CPR for the HETM group compared to the control group (47.4% [18/38] versus 21.3% [10/47], P = 0.019), while we did not see any differences in CPRs by the use of LAH (LAH + ; 36.0% [9/25] and LAH – ; 31.6% [19/60], respectively, p = 0.89) (Fig. 2). The miscarriage rates per clinical pregnancy in the HETM and the control groups were 27.8% [5/18], and 30.0% [3/10], respectively (p = 0.129), while the live birth rate in the HETM group was 34.2% [13/38], which was significantly higher compared to the control group (14.9% [7/47], p = 0.045).

**Table 1:** Clinical outcomes after FET* of euploid blastocysts with good or poor morphology grade#

	Morphologically good blastocyst	Morphologically poor blastocyst	P value
Number of FET*	954	85	
Age, years** [IQR***]	38.0 [35.0–40.0]	39.0 [34.0–40.8]	0.481
Clinical pregnancy, n (%)	628 (65.8)	28 (32.9)	< 0.0001
Miscarriage, n (%, per clinical pregnancy)	73 (11.6)	8 (28.6)	< 0.05
Live birth, n (%, per ET)	555 (58.2)	20 (23.5)	< 0.0001

*FET frozen embryo transfer, **median, ***IQR interquartile range

^#^euploid: except mosaic blastocyst

**Table 2:** Characteristics of morphologically poor euploid FET* with or without HETM

	Control	HETM	P value
Number of FET*	47	38	
Patient age, years** [IQR***]	39.0 [37.0–40.0]	39.0 [34.0–40.8]	0.72
Proportion of AMA^#^ (≥ 40), n (%)	19 (40.4)	16 (42.1)	1.0
Proportion of RIF^##^, n (%)	12 (25.5)	19 (50.0)	0.025
Proportion of RPL^###^, n (%)	0 (0)	1 (2.6)	0.447
Previous ET attempts, n* [range, times]	1 [0–3]	2 [0–5]	0.22
Mode of insemination (IVF/ICSI), n	19/28	8/30	0.065
Endometrial preparation (NOC/HRC)^ǂ^, n	28/19	20/18	0.66
Proportion of LAH^ǂǂ^, n (%)	14 (29.8)	11 (28.9)	1.0

*FET* frozen embryo transfer, **median, **IQR interquartile range

^#^*AMA* advanced maternal age, ^##^ *RIF* repeated implantation failure, ^###^*RPL* recurrent pregnancy loss

^ǂ^NOC/HRC, natural ovulatory cycle/hormone replacement cycle, ^ǂǂ^*LAH* laser assisted hatching

These results led us to identify predictive factors for achieving a higher chance of pregnancy from FET with morphologically poor euploid blastocyst, by comparing the group of 28 pregnant patients with the group of 57 non-pregnant patients (Table 3). The average age in the pregnant group was slightly lower than in the non-pregnant group (37.5 and 39.0 years, respectively, p = 0.242). There were no significant differences found in either the proportions of advanced maternal age, RIF, PRL, number of previous ET attempts, or insemination method. Comparing the endometrial preparation protocols used before FET, the NOC protocol was used more frequently than the HRC protocol (56.4% and 43.6%, respectively). The NOC protocol was used in 67.9% of the pregnant group, which was higher than the non-pregnant group (50.9%, p = 0.76). The proportion of HRC protocol in the pregnant and non-pregnant groups were 32.1% and 49.1%, respectively, and the difference was not significant (p = 0.12). The proportion of embryos that underwent LAH were also comparable between the pregnant and non-pregnant groups (32.15% and 28.1%, respectively, p = 0.801). Interestingly, the proportion of embryos transferred in the HETM medium was 64.3% in the pregnant group, which was significantly higher than in the non-pregnant group (35.1%, p = 0.019).

**Table 3:** Predictive factors for achieving a pregnancy after FET* of morphologically poor euploid blastocysts

	Pregnant patients n = 28	Non-pregnant patients n = 57	P-value	Univariate analysis OR (95%CI)	Multivariate analysis OR (95%CI)
Age, years** [IQR***]	37.5 [34.0–40.0]	39.0 [37.0–40.0]	0.242	0.94 (0.83–1.07)	0.96 (0.77–1.20)
Proportion of AMA^#^ (≥ 40), n (%)	10 (35.7)	25 (43.9)	0.494	0.71 (0.28–1.81)	0.94 (0.19–4.60)
Proportion of RIF^##^, n (%)	8 (28.6)	23 (40.4)	0.343	0.59 (0.22–1.57)	1.02 (0.200–5.14)
Proportion of RPL^###^, n (%)	7 (25.0)	8 (14.0)	0.237	2.04 (0.66–6.34)	2.53 (0.672–9.53)
Previous ET attempts, n [range, times]	0.5 [0.0–4.0]	2.0 [0.0–4.0]	0.087	0.85 (0.71–1.03)	0.83 (0.620–1.10)
Mode of insemination (IVF/ICSI), n	8/20	19/38	0.805	1.25 (0.47–3.36)	0.87 (0.27–2.86)
Endometrial preparation (NOC†/HRC††), n (%)	19/38 (32.1/67.9)	29/28 (50.9/49.1)	0.167	0.49 (0.19–1.27)	0.47 (0.15–1.43)
Proportion of LAH^ǂ^, n (%)	9 (32.1)	16 (28.1)	0.801	1.21 (0.46–3.24)	1.59 (0.50–5.08)
HETM^ǂǂ^, n (%)	18 (64.3)	20 (35.1)	0.019	3.33 (1.29–8.57)	5.08 (1.62–16.0)

*FET frozen embryo transfer, **mean, ***IQR interquartile range

^#^*AMA* advanced maternal age, ^##^*RIF* repeated implantation failures, ^###^*RPL* recurrent pregnancy losses

^†^*NOC* natural ovulatory cycle, ^††^*HRC* hormone replacement cycle

^ǂ^*LAH* laser assisted hatching, ^ǂǂ^*HETM* hyaluronan-enriched transfer medium†

The result from univariate analysis indicated that the use of HETM before embryo transfer increased the chance of positive pregnancy outcomes (OR = 3.33, 95% CI = 1.29–8.57). The multiple logistic regression analysis found that the use of HETM is a predictive factor of positive pregnancy outcomes (OR = 5.08, 95% CI = 1.62–16.0).

## Discussion

The most common way of practice in ART laboratories to select embryos for blastocyst transfer is based on the morphological evaluation by Gardner’s grading method [10]. Timelapse image acquisition systems opened the way to propose alternative methods to select blastocysts for transfer using morphokinetic annotation parameters such as time to reach blastocyst stage, blastocyst expansion speed, ICM and TE grades [10]. In addition to embryo selection methods based on morphology, the increasing use of PGT-A allows clinicians to select blastocysts with a higher probability of implantation based on the chromosomal profiles of the embryo, or euploidy [19, 20]. The potential benefits of combining embryo morphology and PGT-A have been shown in recent publications, where morphological grades could still differentiate the chance of achieving a clinical and ongoing pregnancy among euploid embryos [20, 21]. Our data support these findings: when we compare the transfer of euploid blastocysts based on the morphology grade which was assessed after warming, the clinical pregnancy rate was 32.9% (28/85) for those with poor morphology grade and 65.8% (628/954) for those with good morphology grade (Table 1).

Looking specifically in the FET outcomes of euploid blastocysts with poor morphology grade, embryos in HETM group resulted in much higher success rates than the control group in the same setting. We did not find any significant differences between both groups in terms of patient characteristics, including median age, proportion of advanced maternal age, and proportion of PRL, suggesting that the use of HETM by itself could improve the outcome of FET with morphological poor blastocysts. Although the proportion of ICSI cycles was slightly higher in the HETM group, this difference was not significant. Our study identified a potentially profound impact of endometrial preparation on the pregnancy rates of euploid blastocyst transfer, as the HRC protocol was identified as a negative predictive factor for clinical pregnancy [8]. On the contrary, the proportions of NOC and HRC protocols were quite similar between the control and HETM groups in this study, and there was no bias found regarding endometrial preparation protocols before embryo transfer. Instead, this study showed that the use of HETM in the preparation of blastocyst before transfer improved the clinical pregnancy rate of morphologically poor euploid blastocyst (47.4% vs. 21.3%, p = 0.0194), indicating that hyaluronan supplementation could support better implantation of morphologically poor euploid blastocysts.

Hyaluronan is one of the essential molecules found in key reproductive events including ovulation, fertilization, embryogenesis, implantation and trophoblast invasion [22–24]. Hyaluronan is synthesized as a linear polymer structure of glycosaminoglycan which consists of repeating disaccharide units of D-glucuronic acid and N-acetyl-D-glucosamine, and it was formed in polymer of various molecular sizes up to 10^7^ Da. It is produced predominantly by three isoforms of hyaluronic acid synthase (HAS1-3), each generating polymers of different lengths. Turnover of hyaluronan molecules in physiological environments is relatively short, either by oxidation or enzymatic degradation by a family of hyaluronidases (HYALs) [25]. Among three main isoforms of HYALs, hyaluronidase 2 (HYAL2) is expressed on the cell surface with a glycosylphosphatidylinositol (GPI)-anchor attached to the extracellular side of the plasma membrane [25, 26]. The biological functions of hyaluronan polymers in extracellular matrix are modulated through interaction with a variety of hyaluronan-binding proteins, including the most notable hyaluronan-receptor, CD44 [24, 25, 27]. HYAL-2 is involved in the processing of long chain hyaluronan in the extracellular space through the interaction with CD44, and the cleavage of hyaluronan via HYAL-2/CD44 complex is essential to the subsequent receptor-mediated internalization of hyaluronan into the cytoplasm [28]. Kong et al. recently demonstrated that successful transition of uterine endometrium for establishment of pregnancy requires selective elimination of pro-inflammatory senescent decidual cells by uterine natural killer cells [29]. They also reported that morphologically poor human blastocysts derived from IVF/ICSI express lower levels of HYAL2 compared to morphologically good blastocysts (Gardner’s classification; ≥ BB, p = 0.003). Moreover, successfully implanted blastocysts had higher level of HYAL2 expression compared to blastocysts that failed to implant after transfer (p = 0.042). This suggests that the morphologically poor euploid blastocysts in our study also have lower levels of HYAL2 on the cell structure, which could limit the interaction with CD44 to process and internalize extracellular hyaluronan [26, 29]. Although the information of average molecular weight of hyaluronan in EmbryoGlue® is not available, the enriched-hyaluronan in this transfer medium might facilitate the CD44-mediated internalization process to improve of the implantation of morphologically poor euploid blastocysts. An increase of high molecular weight hyaluronan would also increase the density of extracellular matrix to a level closer to uterine endometrium, which could give rise to an immunotolerant reaction via uterine natural-killer cells [29]. These series of factors could confer additive effects to help the implantation of morphologically poor blastocysts.

As has been demonstrated by numerous reports and publications, morphologically poor blastocysts often result in lower implantation rates, even when only euploid embryos are selected for transfer. Our study strongly suggested that HETM used at the time of blastocyst transfer can significantly improve the chance of morphologically poor euploid blastocysts to achieve a clinical pregnancy. Using HETM provides a relatively simple yet powerful tool for clinicians and embryologists when they find that the post-warm morphology of blastocysts is poor, either immediately after warming or during the subsequent post-warm culture. Our own experience as well as previously published data strongly suggest that the concentration of hyaluronan, and possibly the chain length of hyaluronan polymers, can be essential factors that should be considered while selecting the most suitable embryo transfer medium in regard to improving the chance of clinical pregnancy. This prompted us to use EmbryoGlue® as HETM in our study as the hyaluronan concentration data is disclosed by the manufacturer (0.5 mg/ml). While our findings from this study have its own limitation due to relatively small size of patient population, it supports our previous hypothesis that HETM is especially helpful to improve the success rates of those cycles associated with less favorable factors, either in patient characteristics or in embryos to be transferred. A similar study with larger data set may be required to categorically establish a link between hyaluronan composition of transfer medium and the success rates of embryo transfer.

## Data Availability

Data sharing is not applicable to this article as no new data were created or analyzed in this study.
